# Distribution of lipoprotein (a) levels in patients with lower extremity artery disease and their impact on amputation and survival: a retrospective study

**DOI:** 10.1186/s12944-025-02542-5

**Published:** 2025-04-02

**Authors:** Katrin Gebauer, Nasser M. Malyar, Julian Varghese, Holger Reinecke, Tobias J. Brix, Christiane Engelbertz

**Affiliations:** 1 Department of Cardiology I– Coronary and Peripheral Vascular Disease, Heart Failure, University Hospital Muenster, Cardiol, Muenster, 48149 Germany; 2 Institute of Medical Informatics, University of Muenster, Muenster, 48149 Germany; 3 Department of Cardiology I– Coronary and Peripheral Vascular Disease, Heart Failure University Hospital Muenster, Albert-Schweitzer-Campus 1, Geb. A1, Muenster, 48149 Germany

**Keywords:** Lipoprotein (a), Lipid-lowering therapy, Lower extremity artery disease, Low-density lipoprotein cholesterol, Revascularisation, Amputation, Death

## Abstract

**Background:**

Elevated lipoprotein (a) (Lp(a)) is an independent risk factor for lower extremity artery disease (LEAD) with equivocal effect on amputation and mortality. Results regarding aggressive lipid-lowering therapies (LLT) are missing. We examined LEAD patients with Lp(a) measurement and the impact of intensive LLT on amputation and survival.

**Methods:**

Baseline characteristics of 263 LEAD patients with Lp(a) measurement treated in a tertiary hospital from 01/2017 until 01/2022 were recorded. Patients were categorized into three groups according to their Lp(a) values (< 30 mg/dL, 30–90 mg/dL and > 90 mg/dL). Lipid values and LLT were recorded at baseline and during follow-up (median 750 days). Peripheral endovascular revascularizations (EVR), amputations and death during follow-up were analysed.

**Results:**

Of 263 patients, 75% were male, mean age was 67 ± 10 years. Elevated Lp(a) values ≥ 30 mg/dL were found in 32%, 16% had values > 90 mg/dL. Baseline low-density lipoprotein cholesterol (LDL-C) was 89 ± 38 mg/dL, decreasing to 61 ± 30 mg/dL at follow-up, with no difference between Lp(a) groups (63 ± 32 mg/dL vs. 52 ± 23 mg/dL vs. 60 ± 25 mg/dL, *p* = 0.273). Statin dose was intensified more frequently in those with elevated Lp(a) (16% vs. 35% vs. 33%, *p* = 0.005), who also received significantly more often ezetimibe (50% vs. 58% vs. 73%, *p* = 0.028) and proprotein convertase subtilisin/kexin type 9 inhibitors (2% vs. 3% vs. 8%, *p* = 0.043). No difference was seen regarding EVR (91% vs. 95% vs. 90%, *p* = 0.729), amputations (4% vs. 7% vs. 0%, *p* = 0.245) and death (8% vs. 5% vs. 3%, *p* = 0.436).

**Conclusions:**

Aggressive LLT in high-risk LEAD patients with elevated Lp(a) levels enabled LDL-C target achievement in a majority by combination of established lipid-lowering agents. An increase in EVR, amputation or death could not be observed in patients with high Lp(a) levels.

**Supplementary Information:**

The online version contains supplementary material available at 10.1186/s12944-025-02542-5.

## Introduction

Lower extremity artery disease (LEAD) is a manifestation of atherosclerotic cardiovascular disease (ASCVD), affecting over 200 million people worldwide, with its prevalence and incidence increasing with age [1, 2]. It is characterized by stenosis or occlusion of the arterial vasculature in the lower extremities, leading to intermittent claudication or critical limb-threatening ischemia (CLTI). Common cardiovascular risk factors include smoking, diabetes mellitus, arterial hypertension, chronic kidney disease, dyslipidaemia and a family history of ASCVD [3, 4]. LEAD patients are at high risk for cardiovascular (CV) morbidity and mortality as well as major adverse limb events (MALE) including the need for revascularization and/or amputation, contributing to poor clinical outcomes [5, 6]. The frequent coexistence of multisite atherosclerosis, such as coronary artery disease (CAD) or cerebrovascular disease, further exacerbates the unfavourable prognosis of LEAD patients [7, 8].

Lipoprotein (a) (Lp(a)) is a low-density lipoprotein (LDL)-like particle composed of apolipoprotein (a) covalently bound to apolipoprotein B-100 (apoB-100) via a disulfide bridge. Epidemiological and genetic studies have demonstrated a causal and continuous association between Lp(a) levels and adverse CV outcomes. Lp(a) exhibits pro-atherogenic and pro-inflammatory properties, contributing to residual CV risk in ASCVD patients [9]. This may be attributed to apolipoprotein (a)-mediated endothelial dysfunction, inflammation and calcification, which are further exacerbated by oxidized lipids [10]. Although the relationship between Lp(a) and CV risk appears linear, a threshold of < 30 mg/dL is generally considered non-pathological [9]. Despite its clinical relevance, Lp(a) is measured infrequently in LEAD patients, even less often than in individuals with a history of myocardial infarction (MI) or ischemic stroke [11, 12].

Large cohort studies have demonstrated an association between elevated Lp(a) levels and incident LEAD or the need for revascularization in symptomatic patients [13–20]. However, data on the impact of Lp(a) on revascularization outcomes, patency rates, lesion severity, amputation risk and CV mortality remain inconclusive, particularly in the context of aggressive lipid-lowering therapies (LLT).

Several studies have reported an increased hazard ratio for lower extremity revascularization or amputation in patients with elevated Lp(a) values [21, 22], while no significant association was observed for major adverse cardiac events (MACE) or CV death [21, 23]. In contrast, other studies have linked elevated Lp(a) levels to a higher incidence of MI, stroke and CV mortality [24], disease progression, or increased restenosis rates [25]. In patients with CLTI, higher Lp(a) levels have been associated with an increased risk of CV events, MALE [18, 26], or a combination of both [27], leading to heterogeneous findings across studies investigation the role of Lp(a) in residual CV risk. Notably, all these studies shared a common limitation: suboptimal LLT, characterized by low statin utilization, failure to achieve LDL-cholesterol (LDL-C) target levels, or both.

In this study we analysed LEAD patients with documented Lp(a) levels to assess whether elevated Lp(a) influenced LLT intensification and LDL-C target achievement, and how these factors related to revascularisation rates, amputation risk and overall survival.

## Patients and methods

### Study population and data collection

We retrospectively extracted data from the medical information system of the University Hospital Muenster of 263 inpatients and outpatients aged ≥18 years diagnosed with LEAD based on primary International Classification of Diseases (ICD) code I70.2 (peripheral artery disease) and with documented Lp(a) measurements between January 2017 and January 2020. Patients were stratified into three groups according to their Lp(a) levels: < 30 mg/dL, 30–90 mg/dL and > 90 mg/dL.

### Definition and assessment of patient’s characteristics and laboratory values

Baseline demographic and clinical characteristics, including age, sex and comorbidities (CAD, coronary artery bypass graft (CABG), previous MI, cerebrovascular disease, stroke, diabetes mellitus, arterial hypertension, smoking history) were collected. During the follow-up period (February 2020-May 2023, median 750 days), data on endovascular revascularization procedures, amputations and mortality were recorded.

Lipid profiles and additional blood parameters were documented at baseline, as detailed in the Supplementary Materials. Follow-up LDL-C values were retrieved. The collected blood parameters included haemoglobin (g/dL), leukocyte count (10^3^/mL), platelet count (10^3^/mL), thyroid-stimulating hormone (mU/L), serum creatinine (mg/dL), estimated glomerular filtration rate (eGFR; mL/min/1.73 m^2^), total cholesterol (TC; mg/dL), high-density lipoprotein cholesterol (HDL-C; mg/dL), directly measured LDL-C (mg/dL) and triglycerides (mg/dL).

Lp(a) levels in plasma were determined using a particle enhanced immunoturbidimetric assay on a Cobas c702 automated clinical chemistry analyser (Roche Diagnostics GmbH, Mannheim, Germany) following the manufacturer´s standard protocol. The assay was validated through regular reference sample analyses provided by the German INSTAND proficiency testing program and the international quality assurance program of the US Centers for Disease Control and Prevention. Although Lp(a) was measured in nmol/l, values were converted to mg/dL for better comparability using the equation: mg/dL = (nmol/L + 3.83) × 0.4587.

Chronic kidney disease was classified according to the Kidney Disease: Improving Global Outcomes (KDIGO) criteria based on eGFR values.

- Stage 1: ≥ 90 mL/min/1.73 m^2^.
- Stage 2: 60–89 mL/min/1.73 m^2^.
- Stage 3a: 45–59 mL/min/1.73 m^2^.
- Stage 3b: 30–44 mL/min/1.73 m^2^.
- Stage 4: 15–29 mL/min/1.73 m^2^.
- Stage 5: < 15 mL/min/1.73 m^2^.

For statistical analysis, all data were anonymized. The study was approved by the Ethics Committee of the University of Muenster (reference number 2024-568-f-S).

### Definition of medical therapy

Lipid-lowering therapy at baseline and follow-up included statins (atorvastatin, simvastatin, rosuvastatin, pravastatin, fluvastatin, lovastatin), ezetimibe, bempedoic acid and proprotein convertase subtilisin/kexin type 9 inhibitor (PCSK9i). Daily statin doses at hospital admission or initial outpatient presentation in the angiology department as well as subsequent modifications at discharge were recorded.

Atorvastatin and rosuvastatin were classified as high-intensity statins due to their ability to reduce LDL-C levels by > 50% at moderate-to-high doses. The following dosages were considered appropriate for high-intensity LLT:

- Atorvastatin 40 mg and 80 mg.
- Rosuvastatin 20 mg and 40 mg (highest approved doses).

Statin-naïve patients were defined as those without prior statin therapy. Patients receiving a combination of statins, ezetimibe and bempedoic acid were classified as receiving triple oral LLT.

### LLT intensification in patients not meeting LDL-C targets

Patients with LDL-C levels > 55 mg/dL underwent a LLT intensification according to the current guidelines of the European Society of Cardiology/European Atherosclerosis Society (ESC/EAS) for the management of dyslipidaemias [28] and the German Society of Lipidology (DGFL) [29] stepwise treatment algorithm (Supplementary Figure S1) under the supervision of the treating physician in our hospital.

At follow-up, the proportion of patients achieving LDL-C target level of < 55 mg/dL and < 70 mg/dL was assessed. Additionally, the use of antiplatelet agents (acetylsalicylic acid (ASA), clopidogrel, ticagrelor, prasugrel, ASA combinations) and anticoagulants (phenprocoumon, apixaban, rivaroxaban, edoxaban, dabigatran) was documented.

Patients with missing baseline LLT were excluded from the analysis.

### Statistical analysis

Statistical analyses were performed using IBM SPSS Statistics Version 29.0.0.0 (241). Comorbidities and LLT use are presented as absolute numbers and percentages. Laboratory values are expressed as means with standard deviations (SD).

Differences in categorical variables between the Lp(a) groups as well as statin prescription distributions at admission and discharge were analysed using the Chi Square test with statistical significance set at *p* < 0.05. Differences in continuous variables, such as LDL-C levels at baseline, were assessed using one-way analysis of variance (ANOVA F-test), with *p* < 0.05 considered statistically significant.

Univariate predictors of mortality during follow-up were evaluated using Cox regression analysis and hazard ratios (HRs) with 95% confidence intervals (CI) were calculated. Multivariate Cox regression analyses was performed to identify independent predictors of mortality, adjusting for potential covariates, including in a first analysis the baseline characteristics Lp(a) category, sex, age, arterial hypertension, diabetes mellitus, cerebrovascular disease, CAD and smoking history, and in a second analysis the baseline characteristics Lp(a) category, sex, age, arterial hypertension, diabetes mellitus, cerebrovascular disease, CAD, active smoking, and the follow-up medication high-intensity statin, ezetimibe, antiplatelets and anticoagulants.

## Results

Among the 263 patients included in the study, 75% were male with a mean age was 67±10 years. Inpatient care was required for 67% of the cohort, while 33% were treated as outpatients.

Based on Lp(a) levels, patients were categorized into three groups: 179 (68%) individuals had Lp(a) < 30 mg/dL, 43 (16%) had Lp(a) levels between 30 and 90 mg/dL and 41 (16%) had Lp(a) > 90 mg/dL. Comorbidities, such as CABG, MI, cerebrovascular disease, stroke, diabetes mellitus, arterial hypertension and smoking history were comparably distributed across the Lp(a) groups. However, patients in the highest Lp(a) category (> 90 mg/dL) exhibited a significantly higher prevalence of CAD compared to those with Lp(a) levels of 30–90 mg/dL and < 30 mg/dL, respectively (61% vs. 44% vs. 41%, *p* = 0.006). The distribution of chronic kidney disease stages, classified according to the KDIGO guidelines, was similar across the groups (Table 1).

**Table 1:** Baseline characteristics

	All	Lp(a) < 30 mg/dL	Lp(a) 30–90 mg/dL	Lp(a) > 90 mg/dL	*p*-value
Patients, n (%)	263 (100)	179 (68)	43 (16)	41 (16)	
Age, mean ± SD (years)	67 ± 10	67 ± 10	67 ± 12	65 ± 10	0.647
Male sex, n (%)	198 (75)	136 (76)	36 (84)	26 (63)	0.091
CAD, n (%)	118 (45)	74 (41)	19 (44)	25 (61)	0.006
1-vessel, n (%)	103 (39)	64 (36)	14 (33)	25 (61)	
2-vessel, n (%)	4 (2)	4 (2)	0 (0)	0 (0)	
3-vessel, n (%)	11 (4)	6 (3)	5 (12)	0 (0)	
CABG, n (%)	40 (15)	23 (13)	7 (16)	10 (24)	0.175
MI, n (%)	57 (22)	32 (18)	11 (26)	14 (34)	0.059
Cerebrovascular disease, n (%)	74 (28)	46 (26)	12 (28)	16 (39)	0.231
Stroke, n (%)	40 (15)	30 (17)	5 (12)	5 (12)	0.591
Diabetes mellitus, n (%)	69 (26)	51 (29)	13 (30)	5 (12)	0.082
Arterial hypertension, n (%)	224 (85)	153 (86)	38 (88)	33 (81)	0.585
Nicotine user, n (%)	217 (83)	149 (83)	34 (79)	34 (82)	0.245
active, n (%)	99 (38)	70 (39)	10 (23)	19 (46)	
former, n (%)	118 (45)	79 (44)	24 (56)	15 (37)	
CKD KDIGO stages^$^					0.673
Stage 1, n (%)	0	0	0	0	
Stage 2, n (%)	97 (65)	63 (64)	17 (63)	17 (71)	
Stage 3a, n (%)	30 (20)	22 (22)	4 (15)	4 (17)	
Stage 3b, n (%)	15 (10)	8 (8)	4 (15)	3 (13)	
Stage 4, n (%)	5 (3)	3 (3)	2 (7)	0 (0)	
Stage 5, n (%)	3 (2)	3 (3)	0 (0)	0 (0)	

^$^*n* = 150

CABG = coronary artery bypass graft; CAD = coronary artery disease; CKD = chronic kidney disease; KDIGO = Kidney Disease: Improving Global Outcomes; MI = myocardial infarction; SD = standard deviation

### Baseline laboratory parameters

Baseline haemoglobin and platelets counts showed no significant differences between Lp(a) groups, nor did serum creatinine and eGFR. Leucocyte counts increased with higher Lp(a) levels (8.5±2.2 vs. 9.3±3.5 vs. 9.9±5.9 Thsd./µL, respectively), although this trend narrowly misse statistical significance (*p* = 0.057, Supplementary Table S1).

### Lipid profile at baseline

The mean Lp(a) concentration in the entire cohort was 37±53 mg/dL, with significant differences among the three Lp(a) groups (Table 2).

**Table 2:** Laboratory values

	All *n* = 263	Lp(a) < 30 mg/dL *n* = 179	Lp(a) 30–90 mg/dL *n* = 43	Lp(a) > 90 mg/dL *n* = 41	*p*-value
Lp(a), mg/dL, mean ± SD	37 ± 53	9 ± 7	55 ± 17	140 ± 54	< 0.001
Lp(a), nmol/L, mean ± SD	80 ± 117	17 ± 15	120 ± 38	307 ± 119	< 0.001
Total cholesterol, mg/dL, mean ± SD	160 ± 48	160 ± 48	156 ± 43	162 ± 33	0.805
LDL-C direct, mg/dL, mean ± SD	89 ± 38	89 ± 40	84 ± 35	91 ± 32	0.681
HDL-C, mg/dL, mean ± SD	51 ± 16	51 ± 17	49 ± 14	55 ± 16	0.175
Triglycerides, mg/dL, mean ± SD	153 ± 91	159 ± 97	153 ± 90	125 ± 57	0.099
LDL-C < 55 mg/dL, n (%)	42 (16)	29 (16)	8 (19)	5 (12)	0.714
LDL-C < 70 mg/dL, n (%)	89 (34)	63 (36)	16 (37)	10 (24)	0.353

HDL-C = high-density lipoprotein cholesterol; LDL-C = low-density lipoprotein cholesterol; SD = standard deviation

The mean LDL-C (directly measured) was 89±38 mg/dL, with no significant variation across Lp(a) categories. TC and HDL-C were comparable across Lp(a) groups, while triglyceride levels were numerically lower in patients with Lp(a) > 90 mg/dL. LDL-C target achievement rates were 16% for < 55 mg/dL and 34% for < 70 mg/dL (Table 2).

### LLT at baseline

At baseline, 95% of the patients were receiving statin therapy, with atorvastatin being the most frequently prescribed (62%), followed by rosuvastatin (16%) and simvastatin (14%). High-intensity statins (rosuvastatin ≥20 mg or atorvastatin ≥40 mg) were administered in 60% of the cases. Patients with Lp(a) levels > 90 mg/dL were significantly more likely to receive ezetimibe (58%) compared to those with Lp(a) levels of 30–90 mg/dl (30%) and < 30 mg/dL (35%, *p* = 0.015). The use of bempedoic acid and PCSK9i was infrequent, with rates of 3% and 2%, respectively. Statin dose intensification was initiated in 22% of the patients with LDL-C levels above target, occurring significantly more often in those with moderately (35%) and markedly elevated Lp(a) levels (33%) compared to those with Lp(a) < 30 mg/dL (16%, *p* = 0.005) (Table 3).

**Table 3:** Medication at baseline (a) and medication at follow-up (b) and outcome (c)

(a) Baseline	All n = 263	Lp(a) < 30 mg/dL n=179	Lp(a) 30–90 mg/dL n = 43	Lp(a) > 90 mg/dL n = 41	*p*-value
Statins, n (%)	248 (95)	168 (94)	42 (98)	38 (95)	0.330
Simvastatin, n (%)	37 (14)	24 (13)	7 (16)	6 (15)	
Atorvastatin, n (%)	161 (62)	107 (60)	30 (70)	24 (60)	
Rosuvastatin, n (%)	43 (16)	31 (17)	4 (9)	8 (20)	
Pravastatin, n (%)	6 (2)	6 (3)	0 (0)	0 (0)	
Fluvastatin, n (%)	1 (0.4)	0 (0)	1 (2)	0 (0)	
Ezetimibe, n (%)	98 (37)	62 (35)	13 (30)	23 (58)	0.015
Bempedoic acid, n (%)	4 (3)	2 (3)	2 (10)	0 (0)	0.178
PCSK9i, n (%)	4 (2)	2 (1)	0 (0)	2 (5)	0.160
High-intensity statin, n (%)	157 (60)	106 (59)	26 (61)	25 (61)	0.973
Statin dose intensification, n (%)	57 (22)	29 (16)	15 (35)	13 (33)	0.005
Antiplatelet therapy, n (%)	231 (88)	159 (89)	36 (84)	36 (90)	0.916
ASA, n (%)	60 (23)	40 (22)	11 (26)	9 (23)	
Clopidogrel, n (%)	108 (41)	78 (44)	13 (30)	17 (43)	
ASA + Clopidogrel, n (%)	56 (21)	37 (21)	10 (23)	9 (23)	
ASA + Ticagrelor, n (%)	2 (0.4)	2 (1)	0 (0)	0 (0)	
ASA + Prasugrel, n (%)	5 (2)	2 (1)	2 (4)	1 (3)	
(b) Follow-up	All n = 242	Lp(a) < 30 mg/dL n = 166	Lp(a) 30–90 mg/dL n = 38	Lp(a) > 90mg/dL n = 38	*p*-value
Statin, n (%)	236 (98)	160 (96)	38 (100)	38 (100)	0.133
Simvastatin, n (%)	15 (6)	13 (8)	1 (3)	1 (3)	
Atorvastatin, n (%)	143 (60)	99 (60)	22 (60)	22 (60)	
Rosuvastatin, n (%)	75 (31)	47 (28)	15 (40)	13 (34)	
Pravastatin, n (%)	3 (1)	1 (1)	0 (0)	2 (5)	
High-intensity statin, n (%)	183 (76)	119 (72)	32 (84)	32 (84)	0.109
Ezetimib, n (%)	133 (55)	83 (50)	22 (58)	28 (73)	0.028
Bempedoic acid, n (%)	18 (7)	11 (7)	1 (3)	6 (16)	0.071
PCSK9i, n (%)	8 (3)	4 (2)	1 (3)	3 (8)	0.043
Triple Oral LLT, n (%)	16 (6)	9 (5)	1 (2)	6 (15)	0.037
LDL-C, mg/dL, mean ± SD^#^	61 ± 30	63 ± 32	52 ± 23	60 ± 25	0.273
LDL-C < 55 mg/dL, n (%)^#^	79 (49)	55 (50)	13 (54)	11 (41)	0.595
LDL-C < 70 mg/dL, n (%)^#^	121 (75)	81 (74)	20 (83)	20 (74)	0.603
(c) Outcome	All n = 263	Lp(a) < 30 mg/dL n = 179	Lp(a) 30–90 mg/dL n = 43	Lp(a) > 90 mg/dL n = 41	*p*-value
Revascularisation, n (%)	240 (91)	162 (91)	41 (95)	37 (90)	0.729
n = 0	23 (9)	17 (10)	2 (5)	4 (10)	
n = 1–2	134 (51)	92 (51)	24 (56)	18 (44)	
n = ≥ 3	106 (40)	70 (40)	17 (40)	19 (46)	
Amputation, n (%)	10 (4)	7 (4)	3 (7)	0 (0)	0.245
Death, n (%)	16 (7)	13 (8)	2 (5)	1 (3)	0.436

^#^*n* = 161

ASA = acetylsalicylic acid; LDL-C = low-density lipoprotein cholesterol; LLT = lipid-lowering therapy; PCSK9i = proprotein convertase subtilisin/kexin type 9 inhibitor

### Antiplatelets and anticoagulants at baseline

Antiplatelet therapy was prescribed to 88% of the patients, with clopidogrel being the most commonly used agent (41%), followed by ASA (23%) and a combination of ASA with another agent (21%). Anticoagulant therapy was administered to 32% of the patients. The distribution of antiplatelet and anticoagulant use did not differ significantly among the Lp(a) groups (Supplementary Table S2).

### LLT at follow-up and LDL-C target level achievement

At follow-up, 98% of all patients remained on statin therapy, with 76% receiving high-intensity statins at an appropriate dose. Only 15 patients (6%) received simvastatin at follow-up due to either intolerance issues of other more potent statins or individual target level achievement. Ezetimibe use remained significantly more common in patients with Lp(a) > 90 mg/dL (73%) than in those with Lp(a) levels of 30–90 mg/dL (58%) and < 30 mg/dL (50%, *p* = 0.028). The use of bempedoic acid increased to 16% in the highest Lp(a) group compared to 7% and 3% in lower Lp(a) categories, but did not reach statistical significance (*p* = 0.071). PCSK9i use was significantly higher in the highest Lp(a) group (8%) than in the other groups (3% and 2%, respectively, *p* = 0.043). Triple oral LLT (statin, ezetimibe and bempedoic acid, as described by the escalation algorithm, Supplementary Figure S1) was more frequently implemented in patients with Lp(a) > 90 mg/dL (15%) compared to those in the 30–90 mg/dL (2%) and < 30 mg/dL (5%) groups (*p* = 0.037). Among 161 patients with available LDL-C follow-up measurements, the mean LDL-C level decreased to 61±30 mg/dL. LDL-C target achievement rates improved, with 49% reaching < 55 mg/dL and 75% achieving < 70 mg/dL in 75%, with no significant difference between the three Lp(a) groups (Table 3).

### Revascularization frequency, amputation and survival at follow-up

**Fig. 1:**
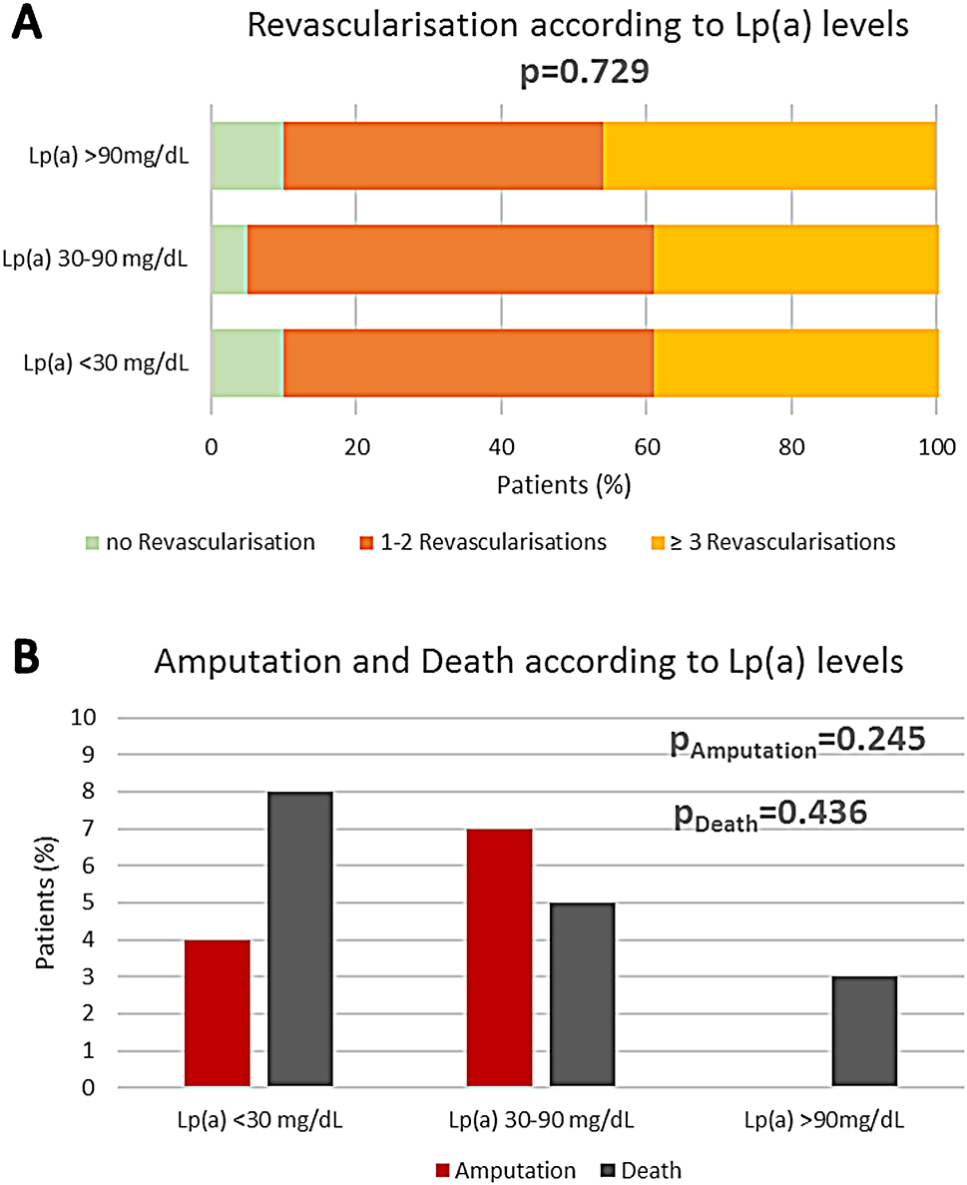
Outcome according to Lp(a) levels. **A**: Revascularisation according to Lp(a) levels. **B**: Amputation (red) and death (grey) according to Lp(a) levels

The overall rate of endovascular revascularizations for LEAD did not differ between the Lp(a) groups. A total of 91% of the patients underwent at least one revascularization procedure after being treated at our facility (Lp(a) < 30 mg/dL: 91% vs. Lp(a) 30–90 mg/dL: 95% vs. Lp(a) > 90 mg/dL: 90%, *p* = 0.7). The majority of patients (51%) underwent one or two endovascular procedures, whereas 40% of the patients in the first two Lp(a) groups and 46% in the highest Lp(a) category had ≥3 revascularizations, though this difference was not statistically significant (Table 3; Fig. 1A).

Amputations was infrequent, occurring in only 10 patients (4%), with no significant difference among the Lp(a) groups (*p* = 0.2). Notably, no amputation was recorded in patients with Lp(a) > 90 mg/dL.

Only 16 subjects (6%) died during follow up of 750 days. Again, there was no significant difference with regard to Lp(a) values, yet numerically most deaths appeared in the Lp(a) group < 30 mg/dL (8%; *p* = 0.4) (Fig. 1B).

In the univariate Cox regression analysis, survival did not differ between patients with Lp(a) values < 30 mg/dL and Lp(a) values ≥ 30 mg/dL (HR 0.471, 95% CI 0.134–1.661, *p* = 0.2), nor among the three Lp(a) categories. In multivariate Cox regression analysis using the baseline parameters as covariates, the only significant predictors of mortality were arterial hypertension (HR 0.216, 95% CI 0.060–0.775, *p* = 0.02) and former smoking (HR 0.075, 95% CI 0.013–0.418, *p* = 0.003). In the second multivariate Cox regression analysis including baseline parameters and medical therapy, arterial hypertension was no longer significant. Age (HR 1.123, 95%CI 1.017–1.239; *p* = 0.02) and active smoking (HR 8.931, 95%CI 1.241–64.287; *p* = 0.03) were associated with higher risk of mortality, whereas treatment with high-intensity statin (HR 0.215, 95%CI 0.039–1.203; *p* = 0.08) showed a tendency to a non-significant association with decreased mortality risk.

## Discussion

This study of 263 LEAD patients with Lp(a) measurement revealed that aggressive LLT resulted in more pronounced LDL-C target achievement by use of established lipid-lowering agents irrespective of Lp(a) levels. Amputation and death were scarce despite this high-risk patient population and did not show an association with Lp(a) levels. To our knowledge, this is the first work to elucidate that LDL-C goal achievement in LEAD can be reached by implementing an individualized therapeutic approach with common secondary prevention drug regimen potentially attenuating the increased risk of amputation and death which might also be influenced by elevated Lp(a).

Antiplatelet therapy in 88% and LLT in 95% at baseline as well as 98% at follow-up with a tremendous proportion of high-intensity statin (76%), ezetimibe (55%) and bempedoic acid (7%) as triple oral LLT may be the tool to decrease adverse outcomes in LEAD.

Even though Lp(a) has been shown to be associated with incident and prevalent LEAD [9, 14, 30], its effect on outcome has been inconsistent. Whereas in some studies Lp(a) was associated with LEAD progression [31], increased restenosis [25], mortality and hospitalization [16], lower limb revascularization [21, 24], limb amputation [22], as well as MACE [23] and MALE [27], other studies were unable to show that Lp(a) levels were predictive of incident LEAD and outcomes in LEAD [20, 21, 32]. This may in part be explained by large heterogeneity of the study populations and in different Lp(a) cut-off values as well as different follow-up periods. All of the studies demonstrating a correlation between Lp(a) levels and outcome had suboptimal LLT and LDL-C levels surmounting the current guideline recommended LDL-C target levels < 55 mg/dL [28] and < 70 mg/dL [33] by far.

### Lp(a) and its effect on MALE and MACE

The endpoint lower limb revascularisation was examined by several large studies reporting different results than observed in our study, but also reporting insufficient LLT. Golledge et al. [21] found that LEAD participants ≥ 72 years of age with Lp(a) levels ≥ 30 mg/dL underwent lower limb revascularization more frequently and had an increased risk of all-cause mortality. However, statin use was 73%, yet LDL-C levels with 93 mg/dL leaving unused potential to improve secondary prevention. Guédon et al. [15] performed a large analysis of more than 16,000 patients and found a significant link between Lp(a) levels and LEAD events including limb revascularization and amputation for ischemia after adjusting for CAD and stroke. However, LDL-C levels were even higher with 118 mg/dL and the proportion of LLT was 53% only.

Also the endpoint limb amputation was analysed by others with opposing results. The prospective FRENA registry demonstrated an increased risk for MI (adjusted HR 5.22; 95% CI 3.06–8.92), stroke (adjusted HR 8.96; 95% CI 4.41–18.21) and limb amputation (adjusted HR 3.20; 95% CI 1.36–7.52) in stable LEAD outpatients with Lp(a) between 30 and 50 mg/dL. Again, overall use of statin was 75%, but LDL-C levels were 115 mg/dL and only 8% reached a target LDL-C < 70 mg/dL [22].

Other studies reported higher supply with statins in patients with LEAD, but without reaching LDL-C target levels and with inconsistent associations of high Lp(a) levels with MACE and MALE. Ezhov et al. [24] found a correlation with Lp (a) > 30 mg/dL in 258 patients with LEAD and CV disease resulting in an increased risk of MACE (HR 3.0; 95% CI 1.5–6.3) and repeated revascularization (HR 2.9; 95% CI 2.0-4.2) after a follow-up period of 3 years despite antiplatelet therapy in 99% and statin medication in 92%. Baseline LDL-C levels were 93 mg/dL and information about LDL-C target level achievement or combination LLT were missing. Another study found no correlation of Lp(a) values and CV death in 1,222 LEAD patients irrespective of 88% statin use and significantly higher ezetimibe application in 11.8% of the patients with highly elevated Lp(a) levels. However, LDL-C target level achievement < 55 mg/dL was 16% and < 70 mg/dL 35%, respectively [23]. This disappointing LDL-C goal achievement is in line with the SANTORINI and DA VINCI registry [34, 35] and can be explained by underuse of add-on LLT in addition to statin medication.

Tomoi et al. [27] found an increased risk for MACE (all-cause death, myocardial infarction, stroke) and MALE (repeat revascularization and amputation) in Lp(a) > 30 mg/dL. However, statin application was present in 62% only with an average LDL-C of 97 mg/dL. Interestingly, in the subgroup analysis, patients with LDL-C < 70 mg/dL were no longer at risk of MACE. The overall independent association with unfavourable outcomes irrespective of LDL-C levels and statin administration may have been caused by a large proportion of patients undergoing dialysis (30%) in contrast to our work (3%), who are at extensively elevated risk of amputation and death per se. Moreover, declining kidney function leads to increased Lp(a) levels [36, 37] and may have contributed to higher acquired Lp(a) levels which may not equally add to the risk of adverse outcomes as life-long elevated Lp(a) values.

In all the above mentioned trials as well as in our study, there was no information on the amount of patients with low molecular weight apo(a) phenotype, which in combination with high Lp(a) concentrations has been shown to increase the risk of atherosclerosis, e.g. coronary heart disease and LEAD [38, 39]. The absence of this measurement might have influenced the results and the drawn conclusions.

### LDL-C target level achievement

In our study, mean Lp(a) levels were 37±53 mg/dL, which appeared higher than those reported in the previously cited studies [15, 21, 24, 27]. Baseline LDL-C was 89±38 mg/dL and was successfully reduced to 61±30 mg/dL at follow-up. The intensification of statin therapy and the frequent use of adjunctive LLT, such as ezetimibe and bempedoic acid, led to an unprecedented LDL-C target level achievement of < 55 mg/dL in 49% and < 70 mg/dL in 75% of the patients. Despite the well-established efficacy of combining statins with ezetimibe and bempedoic acid for LDL-C reduction [40], these strategies have historically been underutilized.

Importantly, intense LDL-C reduction has been shown to improve outcomes in both MACE and MALE [33], primarily through statin therapy. The addition of PCSK9i to high-intensity statins has demonstrated significant benefits in LEAD patients, particularly in reducing the risk of MACE [41] and MALE [42, 43]. However, it remains unclear whether the observed outcome improvements are attributable to the 25–30% Lp(a) reduction achieved with PCSK9-monoclonal antibodies or if these benefits are solely driven by LDL-C lowering. This raises the question of whether rigorous LDL-C management, in combination with comprehensive control of other CV risk factors, can mitigate the residual risk associated with elevated Lp(a).

In our study, patients with extremely high Lp(a) levels were significantly more likely to receive ezetimibe, triple oral LLT and/or PCSK9i. Notably, ezetimibe has been associated with a reduction of MACE in a cohort that included LEAD patients [44]. Furthermore, the anti-inflammatory properties of statins and bempedoic acid may contribute to effective risk reduction in this high-risk population independent of Lp(a) concentrations [45]. Thus, the excess cardiovascular risk associated with elevated Lp(a) may be partially offset by oral LLT particularly through the use of high-potency statins [46].

Ongoing clinical outcomes trials investigating novel Lp(a)-lowering therapies, such as pelacarsen [47] and olpasiran [48], in conjunction with intensive LDL-C reduction will provide further insights into whether targeting Lp(a) specifically offers additional cardiovascular risk reduction beyond LDL-C lowering alone.

### Limitations

This study has several limitations. First, as data were obtained from a single tertiary care centre, selection bias cannot be ruled out.

Second, LLT in patients with LEAD may have been adjusted by primary care physicians or other health providers. Consequently, information on modifications to LLT might be incomplete.

Third, endovascular revascularization procedures and amputation may have been performed at other medical institutions. However, given that patients undergo at least annual follow-up visits at our clinic with regular updates to their medical history, the likelihood of missing data is reduced.

Fourth, the total number of endovascular revascularizations reflects interventions performed over previous time periods when LDL-C target levels may not have been consistently established.

Fifth, Lp(a) levels are known to be inversely correlated with declining renal function, tend to increase with age, particularly in postmenopausal women, and can be reduced by PSCK9i, which was applied in 8 patients (3%) in our study. Therefore, changes in Lp(a) levels cannot be ruled out and reassessment of Lp(a) levels over time might have been necessary to account for potential fluctuations. Furthermore, the absence of information on low molecular weight apo(a) phenotype, which increases the risk of atherosclerosis in patients with high Lp(a) levels, might have biased our results.

Despite these limitations, this study includes a cohort of 263 consecutive LEAD patients with documented Lp(a) measurements, all of whom were referred to a tertiary care centre for specialized treatment by primary care physicians, general practitioners, cardiologists or angiologists. This suggests that the cohort is representative of an unselected real-world patient population.

## Conclusion

In our study, aggressive LLT in high-risk LEAD patients with elevated Lp(a) levels enabled LDL-C target achievement in a majority by combination of established lipid-lowering agents. With extensively lowered LDL-C values, an increase in EVR, amputation or death could not be observed in patients with high Lp(a) levels.

## Electronic supplementary material

Below is the link to the electronic supplementary material.

Supplementary Material 1

## Data Availability

The datasets used and analysed during the current study are available from the corresponding author on reasonable request.

## Abbreviations

ASCVD: Atherosclerotic cardiovascular disease
ASA: Acetylsalicylic acid
CABG: Coronary artery bypass graft
CAD: Coronary artery disease
CI: Confidence interval
CKD: Chronic kidney disease
CLTI: Critical limb-threatening ischemia
CV: Cardiovascular
eGFR: Estimated glomerular filtration rate
EVR: Endovascular revascularization
HDL-C: High-density lipoprotein cholesterol
ICD: International Statistical Classification of Diseases and Related Health Problems
KDIGO: Kidney Disease Improving Global Outcomes
LEAD: Lower extremity artery disease
LDL-C: Low-density lipoprotein cholesterol
LLT: Lipid-lowering therapy
Lp(a): Lipoprotein (a)
MACE: Major adverse cardiovascular events
MALE: Major adverse limb events
MI: Myocardial infarction
PCSK9i: Proprotein convertase subtilisin/kexin type 9 inhibitor
SD: Standard deviation
TC: Total cholesterol
Thsd: Thousand
