# Supplementary material for: Distribution of lipoprotein (a) levels in patients with lower extremity artery disease and their impact on amputation and survival: a retrospective study
Source: Lipids Health Dis. 2025 Apr 2;24:128. doi: 10.1186/s12944-025-02542-5 (PMC11963429; doi:10.1186/s12944-025-02542-5)
Supplement: Supplementary file 1 — Supplementary Material 1 [file 12944_2025_2542_MOESM1_ESM.pdf]

Supplements to

Distribution of lipoprotein (a) levels in patients with lower extremity artery disease and their impact on amputation and survival: a retrospective study

Katrin Gebauer[a], Nasser M. Malyar[a], Julian Varghese[b], Holger Reinecke[a], Tobias J. Brix[b], Christiane Engelbertz[a]

[a] Department of Cardiology I – Coronary and Peripheral Vascular Disease, Heart Failure, University Hospital Muenster, Cardiol, 48149 Muenster, Germany

[b] Institute of Medical Informatics, University of Muenster, 48149 Muenster, Germany

Content

Supplementary Table S1: Laboratory values

Supplementary Table S2: Oral anticoagulation at baseline

Supplementary Figure S1: Escalation strategy of lipid-lowering therapy

Supplementary Table S1: Laboratory values

	All<br>n=263	Lp(a)<br><30 mg/dL<br>n=179	Lp(a)<br>30-90 mg/dL<br>n=43	Lp(a)<br>>90 mg/dL<br>n=41	*p*-value
Serum-Creatinin[+], mg/dL, mean±SD	1.1±0.5	1.1±0.5	1.2±0.4	1.0±0.3	0.161
eGFR ml/min/1.73m^2, mean±SD	65±19	66±19	61±20	69±17	0.399
Haemoglobin, g/dL, mean±SD	13.3±2	13.3±2	13.1±2.2	13.5±1.7	0.712
Leucocytes, Thsd./µL, mean±SD	8.8±3.3	8.5±2.2	9.3±3.5	9.9±5.9	0.057
Platelets, Thsd./µL, mean±SD	240±75	237±70	240±103	252±61	0.613
TSH[§], µU/mL, mean±SD	1.8±2.1	1.8±2.3	1.9±1.5	1.5±1.0	0.790

[+] n=211

[§] n=194

eGFR=estimated glomerular filtration rate; SD=standard deviation; TSH=thyroid stimulating hormone.

Supplementary Table S2: Oral anticoagulation at baseline

	All<br><br>n=263	Lp(a)<br><30 mg/dL<br>n=179	Lp(a)<br>30-90 mg/dL<br>n=43	Lp(a)<br>>90 mg/dL<br>n=41	*p*-value
Anticoagulants, n (%)	85 (32)	55 (31)	16 (37)	14 (35)	0.372
Phenprocoumon, n (%)	18 (7)	12 (7)	3 (7)	3 (8)	
Apixaban, n (%)	35 (13)	20 (11)	9 (21)	6 (15)	
Rivaroxaban, n (%)	24 (9)	17 (10)	2 (5)	5 (13)	
Edoxaban, n (%)	7 (3)	6 (3)	1 (2)	0 (0)	
Dabigatran, n (%)	1 (0.4)	0 (0)	1 (2)	0 (0)	

Supplementary Figure S1: Escalation algorithm of lipid lowering therapy

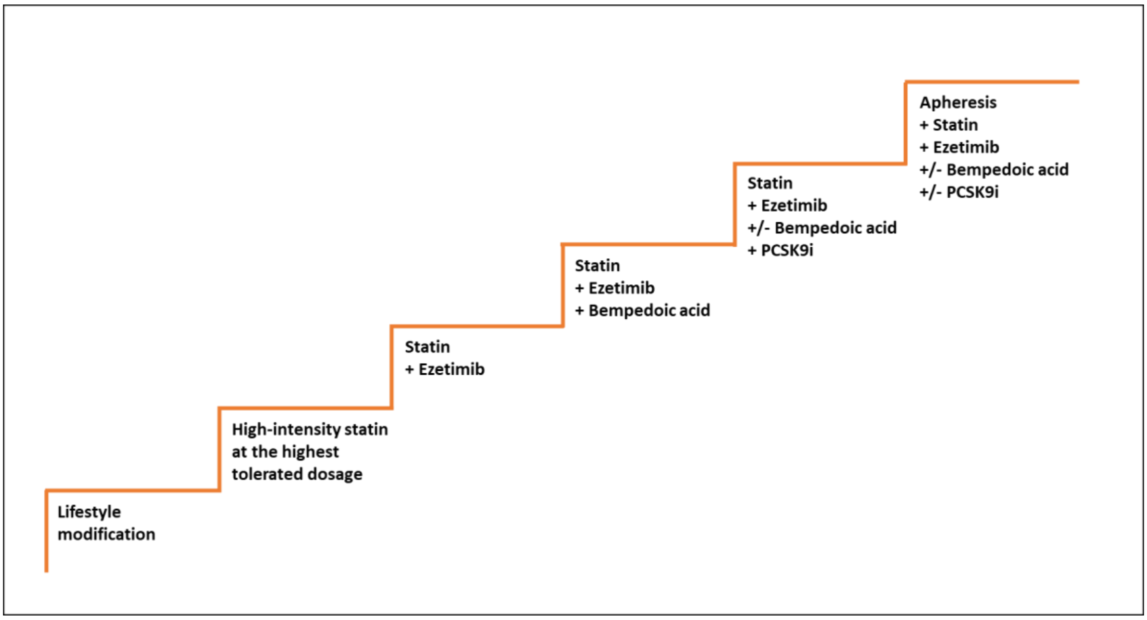

The escalation algorithm of lipid-lowering therapy starts with lifestyle modifacations to lower low-density lipid cholersterol (LDL-C) levels. If target LDL-C level is not met, high-intensity statin at the highest tolerated dosage should be added. If LDL-C target level is still not met, the lipid-lowering therapy should be escalated by consecutive addition of ezetimib, bempedoic acid and proprotein convertase subtilisin/kexin type 9 inhibitors (PCSK9i) as pictured above. The escalation scheme is adopted from the German Society of Lipidology (Deutsche Gesellschaft für Lipidologie e.V. – Lipid-Liga. Empfehlungen zur Diagnostik und Therapie von Fettstoffwechselstöfungen. https://www.lipid-liga.de/empfehlungen/. Accessed 11 Feb 2025.)
